# Unearthing of the Antidiabetic Potential of Aqueous Extract of *Solanum betaceum* Cav. Leaves

**DOI:** 10.3390/molecules28083291

**Published:** 2023-04-07

**Authors:** Raquel Martins, Fátima Fernandes, Patrícia Valentão

**Affiliations:** REQUIMTE/LAQV, Laboratório de Farmacognosia, Departamento de Química, Faculdade de Farmácia, Universidade do Porto, Rua de Jorge Viterbo Ferreira n° 228, 4050-313 Porto, Portugal; raquel2001martins@gmail.com

**Keywords:** *S. betaceum* leaves, phenolic profile, diabetes, oxidative stress

## Abstract

*Solanum betaceum* Cav., commonly known as tamarillo or Brazilian tomato, belongs to the Solanaceae family. Its fruit is used in traditional medicine and food crops due to its health benefits. Despite the numerous studies involving the fruit, there is no scientific knowledge about the tamarillo tree leaves. In this work, the phenolic profile of aqueous extract obtained from *S. betaceum* leaves was unveiled for the first time. Five hydroxycinnamic phenolic acids were identified and quantified, including 3-*O*-caffeoylquinic acid, 4-*O*-caffeoylquinic acid, chlorogenic acid, caffeic acid and rosmarinic acid. While the extract displayed no effect on α-amylase, the extract inhibited the activity of α-glucosidase (IC_50_ = 1617 mg/mL), and it was particularly effective for human aldose reductase (IC_50_ = 0.236 mg/mL): a key enzyme in glucose metabolism. Moreover, the extract exhibited interesting antioxidant properties, such as a potent capacity to intercept the in vitro-generated reactive species O_2_^•−^ (IC_50_ = 0.119 mg/mL) and ^•^NO (IC_50_ = 0.299 mg/mL), as well as to inhibit the first stages of lipid peroxidation (IC_50_ = 0.080 mg/mL). This study highlights the biological potential of *S. betaceum* leaves. The scarcity of research on this natural resource underscores the need for additional studies in order to fully explore its antidiabetic properties and to promote the value of a species currently at risk of extinction.

## 1. Introduction

Natural products have been essential in the discovery of many drugs and are the best options for identifying novel agents and active templates [1]. Over the years, plant extracts have been used on a large scale in both the prevention and treatment of a large spectrum of diseases, including inflammation, diabetes and neurodegenerative disorders [2]. *Solanum betaceum* Cav. (Solanaceae) (synonyms: *Cyphomandra betacea* (Cav.) Sendtn., *Cyphomandra betacea* var. *betacea*, *Cyphomandra crassifolia* (Ortega) J.F. Macbr., *Cyphomandra procera* Wawra, *Pionandra betacea* (Cav.) Miers, *Solanum crassifolium* Ortega, *Solanum insigne* Lowe and *Solanum obliquum* Bertero ex Dunal, nomen nudum)*,* commonly known as tamarillo or Brazilian tomato tree, belongs to the Solanaceae family. The numerous species of this family are distributed in tropical and subtropical areas across the Earth. It is native to the South American region, including Argentina, Bolivia, Chile, Ecuador and Peru, and was later distributed to other countries around the world, such as Portugal [3]. However, this species is classified as vulnerable and appears in the Red List of species at high risk of extinction in the wild according to the International Union for Conservation of Nature and Natural Resources [4]. Tamarillo, the *S. betaceum* fruit, is used in folk medicine and food crops, and several benefits to human health have been reported. The fruit has been the object of several studies due to its biological properties, including antioxidant, anti-inflammatory, anticancer, anti-obesity and antinociceptive activities, and its chemical composition is already well known [5,6]. However, scientific knowledge of the chemical composition and the biological properties of the *S. betaceum* leaves is scarce. As far as we know, only one study that reported the seasonal accumulation of mineral nutrients by tamarillo tree leaves is known [7].

Diabetes is considered an important public health problem, and it is one of the four priority noncommunicable diseases targeted for action by world leaders. Regardless of the vast number of drugs available for diabetes mellitus treatment, the number of cases and their prevalence have been increasing over the past few decades. In 2019, the global age-standardized point prevalence and death rates for type 2 diabetes increased by 49% and 10.8%, respectively, compared to 1990 [8,9]. Diabetes mellitus type 2 has been associated with insulin resistance and/or a change in insulin secretion in cases where pancreatic β-cells are unable to produce insulin. Both conditions result in a hyperglycemic state, which is the main pathophysiological feature of diabetes.Insulin resistance has a complex pathophysiology that can be explained by many factors. One key factor is the intracellular signal pathway converging on the Insulin Receptor Subtract (IRS). Free radicals and oxidative stress are among the major mediators of autoimmune destruction of beta cells in type 1 diabetes or beta cell malfunction and death caused by glucotoxicity and insulin resistance in type 2 diabetes [10]. The main types of reactive species generated in living systems include superoxide anion radical (O_2_^•−^), nitric oxide radical (^•^NO), hydroxyl radical, hydrogen peroxide and singlet radical species [10].

There are many targets underlying metabolic changes in diabetes which constitute possible therapeutics for this disease, namely the ability to modulate the activity of enzymes involved in the metabolism of carbohydrates (α-amylase and α-glucosidase) and glucose (aldose reductase) and the capacity to manage oxidative stress [11].

Given the growing awareness of the role of natural products in disease prevention, relief and treatment and the absence of scientific knowledge about *S. betaceum* leaves, this work aimed to provide knowledge and promote the value of this still-unexplored natural matrix. In line with the goal to minimize the negative environmental impacts resulting from the use of organic solvents, water was selected as the extractor solvent, as it is non-toxic, environmentally friendly, cost-effective and abundant and possesses a good capacity to extract a wide range of bioactive compounds. Hence, the phenolic compound profile of aqueous extract from *S. betaceum* leaves was established using high-performance liquid chromatography (HPLC) coupled with a diode array detector (DAD). The antidiabetic potential was explored using a panel of in vitro targets underlying metabolic changes in diabetes, namely the capacity to inhibit the activity of enzymes involved in the metabolism of glucose (aldose reductase) and carbohydrates (α-glucosidase and α-amylase) and to manage oxidative stress through a nitric oxide and superoxide anion radical scavenging ability and lipid peroxidation protection.

## 2. Results and Discussion

### 2.1. Phenolic Compound Profile

**Figure 1:**
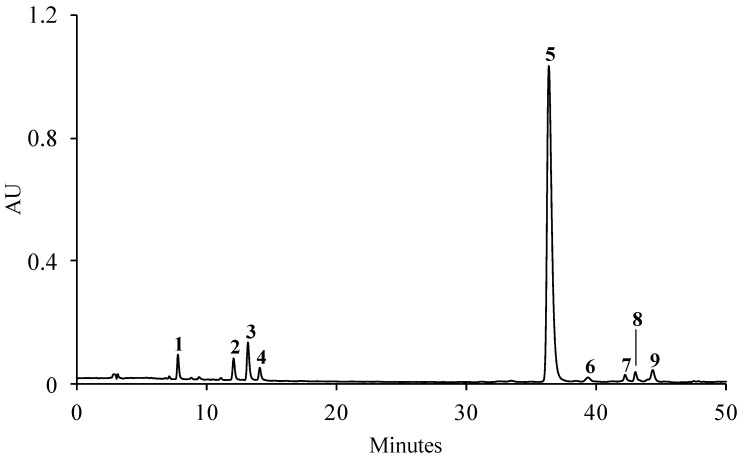
Representative HPLC-DAD chromatogram of the phenolic profile of the aqueous extract of *S. betaceum* leaves detected at 320 nm. (**1**) 3-*O*-Caffeoylquinic acid, (**2**) 4-*O*-caffeoylquinic acid, (**3**) chlorogenic acid, (**4**) caffeic acid, (**5**) rosmarinic acid, (**6** and **9**) unknown kaempferol glycoside derivatives, (**7** and **8**) unknown hydroxycinnamic acid derivatives.

The HPLC-DAD analysis of the aqueous extract of *S. betaceum* leaves revealed the presence of nine phenolic compounds, including seven hydroxycinnamic acids (compounds **1**–**5**, **7** and **8**) and two flavonoids (compounds **6** and **9**) (Figure 1). As far as we know, the phenolic profile of *S. betaceum* leaves was established here for the first time. The sample presented a phenolic profile mainly constituting caffeic-acid-related compounds, namely 3-*O*-caffeoylquinic (**1**), 4-*O*-caffeoylquinic (**2**), chlorogenic (**3**), caffeic (**4**) and rosmarinic (**5**) acids. Although the UV–vis spectra of the compounds **7** and **8** enabled their classification as hydroxycinnamic acids, it was not possible to achieve the complete identification of these compounds. As the compounds **6** and **9** presented UV–vis spectra characteristic of kaempferol glycoside, they were identified as unknown kaempferol glycoside derivatives. Although kaempferol-3-*O*-rutinoside has been described in *S. betaceum* fruits [12,13], this flavanol was not found in the leaves. Among the compounds identified here in the leaves, only 3-*O*-caffeoylquinic (**1**), chlorogenic (**3**) and rosmarinic acids (**5**) were shared with *S. betaceum* fruit [12,13].

A total of 121.57 mg of phenolic compounds/g dry extract was obtained, of which rosmarinic acid (**5**) alone accounted for ca. 82% (Table 1). The caffeoylquinic acids, together, represented almost 10% of the total compounds identified, with chlorogenic acid (**3**) found as the most representative caffeoylquinic acid (Table 1).

### 2.2. S. betaceum Leaves Extract in Glycaemic Control

Glycemic control is fundamental in the control and progression of diabetes. α-Glucosidase and α-amylase have the capacity to modulate the metabolism of glucose and carbohydrates, respectively. α-Glucosidase is an exoenzyme that is present in the small intestine and promotes delayed glucose absorption and does not interfere with insulin. Its inhibition has a relevant role in controlling postprandial hyperglycemia [14]. On the other hand, α-amylase is responsible for breaking down α-(1→4)-glycosidic linkages of complex sugars and can be found in saliva and pancreatic juice. Recently, polyphenol-rich functional foods have been proposed to be unique supplementary and nutraceutical treatments for diabetes mellitus. The inhibition of α-amylase and α-glucosidase enzymes using natural products (especially polyphenols) is a novel oral method used to regulate carbohydrate metabolism and hyperglycemia [15].

**Figure 2:**
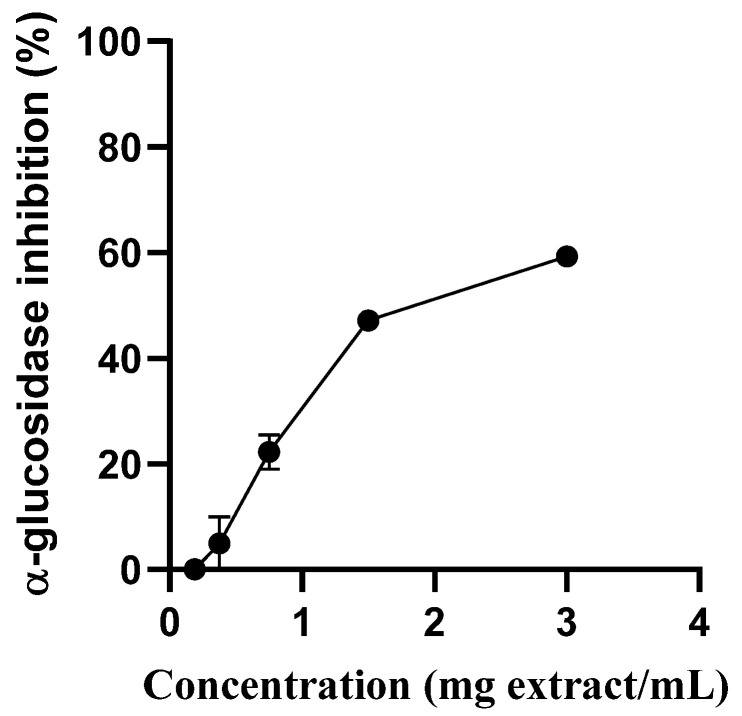
Inhibition of the activity of α-glucosidase by *S. betaceum* aqueous extract. Results are expressed as mean ± SD of three independent experiments, each performed in triplicate.

*S. betaceum* aqueous extract showed an concentration-dependent manner inhibitory capacity towards yeast α-glucosidase (IC_50_ = 1.617 mg/mL) (Figure 2). Therefore, this extract showed the capacity to modulate the mechanism of glucose by slowing down α-glucosidase activity, which has an important role in decreasing postprandial hyperglycemia. According to the guidelines of the International Diabetes Federation [16], α-glucosidase inhibition in combination with other drugs, such as insulin, metformin and sulfonylureas, is the best treatment option for uncontrolled hyperglycemia in diabetic patients. Furthermore, it can be used in overweight diabetes patients once it can help in weight loss. [14]. As previously acknowledged, the sensitivity of the enzymes used in in vitro screening assays can vary easily according to their biological origin (e.g., yeast, rat or human) and acquisition process (native or heterologous expression). While yeast glucosidase and human glucosidase share some similarities in their enzymatic functions, it is not always possible to directly transpose the results obtained in assays using yeast glucosidase to human glucosidase. One reason for this is that the two enzymes have different primary structures and may exhibit different substrate specificities, reaction kinetics and pH and temperature optima. Additionally, they may be subject to different regulatory mechanisms and post-translational modifications that can affect their activity and function [17]. Therefore, it is important to validate the results obtained using yeast glucosidase with human glucosidase to ensure the relevance and accuracy of these findings. Experiments using enzyme-enriched cell supernatants obtained from homogenates of human small intestine cells can be useful for evaluating the effect of the extract on human glucosidase.

In contrast, the extract does not show any inhibitory capacity towards α-amylase. This was an expected behavior, considering the phenolic profile observed was mainly composed of caffeic-acid-related phenols. Chlorogenic acid has been reported to be a weak (or not a strong) inhibitor of α-amylase in the enzyme/inhibitor/starch digestion system. As reported in a recent work [18], caffeoyl substitution significantly decreased α-amylase inhibition of quinic acid through reducing its binding affinity to the enzyme. Furthermore, Tlili and colleagues recently concluded that extracts with high amounts of rosmarinic acid showed a more effective inhibitory capacity towards α-glucosidase than α-amylase, suggesting that this compound could be more effective in inhibiting α-glucosidase [19]. Thus, our results are in harmony with previous data showing a more selective inhibitory capacity of phenolic compounds towards α-glucosidase rather than α-amylase. Additionally, although the effect of an extract cannot simply be extrapolated from the activities of their isolated compounds, synergic effects between rosmarinic, caffeic and caffeoylquinic acids can be suggested as additional factors for the differential α-glucosidase/α-amylase inhibitory effects observed. The major drawback of conventional α-glucosidase inhibitors is their non-selective and strong inhibition of α-amylase. This results in the development of serious gastrointestinal side effects; therefore, pharmacological approaches relying on strong inhibitory actions against α-glucosidase and weak-to-moderate inhibitory properties against α-amylase have been suggested to hold great promise [15].

### 2.3. S. betaceum Leaf Aqueous Extract in the Management of Diabetes-Related Complications

#### 2.3.1. Aldose Reductase Inhibition

Aldose reductase is an enzyme that can reduce glucose to sorbitol in the presence of NADPH by the polyol pathway. The accumulation of sorbitol is implicated in the development of diabetic microvascular lesions such as neuropathy (nervous system), nephropathy (kidneys) and retinopathy (cataracts and glaucoma). Aldose reductase inhibitors are of great importance because they can prevent complications related to diabetes in some tissues in which glucose uptake is independent of insulin. Complications related to the disease are one of the causes of death [20].

**Figure 3:**
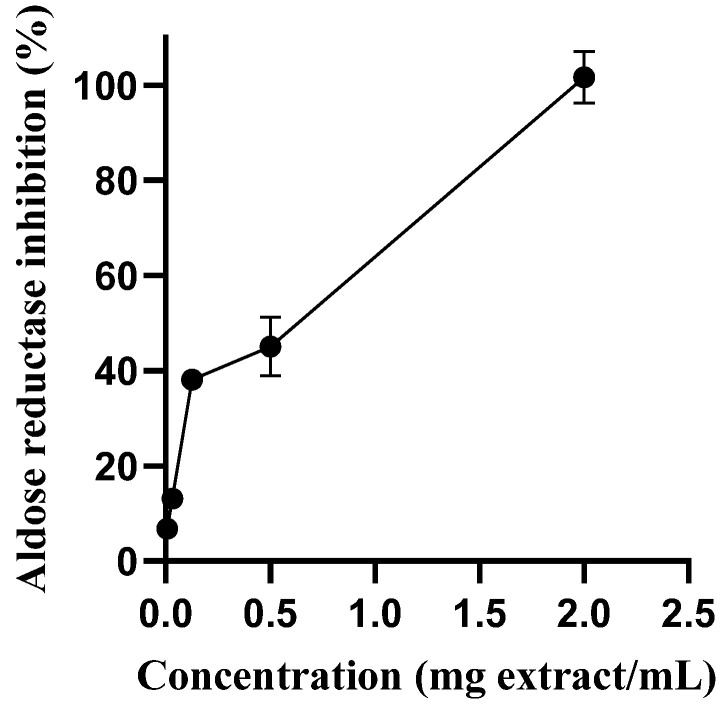
Aldose reductase inhibitory activity of *S. betaceum* leaf aqueous extract. Results are expressed as mean ± SD of six independent experiments, each performed in triplicate.

As seen in Figure 3, the extract was able to inhibit aldose reductase activity in a concentration-dependent manner. It was observed an IC_50_ for aqueous extract only two times higher than that obtained with rutin, the positive control used, IC_50_ = 0.236 mg/mL and IC_50_ = 0.147 mg/mL

This result can be explained by the presence of caffeic-acid-related phenols in the extract. Previous studies have made it possible to classify rosmarinic acid, caffeic acid and 3-, 4- and 5-*O*-caffeoylquinic acids as strong human recombinant aldose reductase inhibitors [21,22]. Indeed, these phenolic compounds, present in high amounts in *S. betaceum* leaf aqueous extract, meet the reported structural requirements for aldose reductase inhibition, including the acidic group that forms an ionic interaction in the binding pocket of the active site of the enzyme and the aromatic moiety which is placed on the lipophilic pocket [17].

Additionally, flavonol glycosides, including kaempferol glycoside derivatives, can also contribute to the observed activity; their protection against diabetes complications by inhibiting the aldose reductase has already been reported [23].

#### 2.3.2. Oxidative Stress

Considerable scientific evidence suggests that hyperglycemia represents the main cause of complications of diabetes, and oxidative stress, resulting from an increased generation of reactive species, plays a crucial role in its pathogenesis [24]. The exacerbated production of free radicals occurring in hyperglycaemia, mainly resultant from nonenzymatic glycation and glucose autoxidation, and the decrease of glutathione levels [25], lead on the intracellular accumulation of reactive oxygen species (ROS), like O_2_^•−^, and of nitrogen-derived radicals (e.g., ^•^NO) and a consequent activation of stress-sensitive intracellular signalling pathways [24]. The latter plays a key role in the development of late complications of diabetes and in mediating insulin resistance (i.e., resistance to insulin-mediated glucose uptake by some cells) and impaired insulin secretion [24]. The simultaneous increase in O_2_^•−^ and ^•^NO generated by hyperglycemia also produces reactive peroxynitrite anions (ONOO^−^), a potent oxidant that oxidizes sulfhydryl groups in proteins and initiates the lipid peroxidation of the biological membranes [26,27]. The targeting of oxidative stress has been highlighted as a method of great promise for managing diabetes and its related complications.

**Figure 4:**
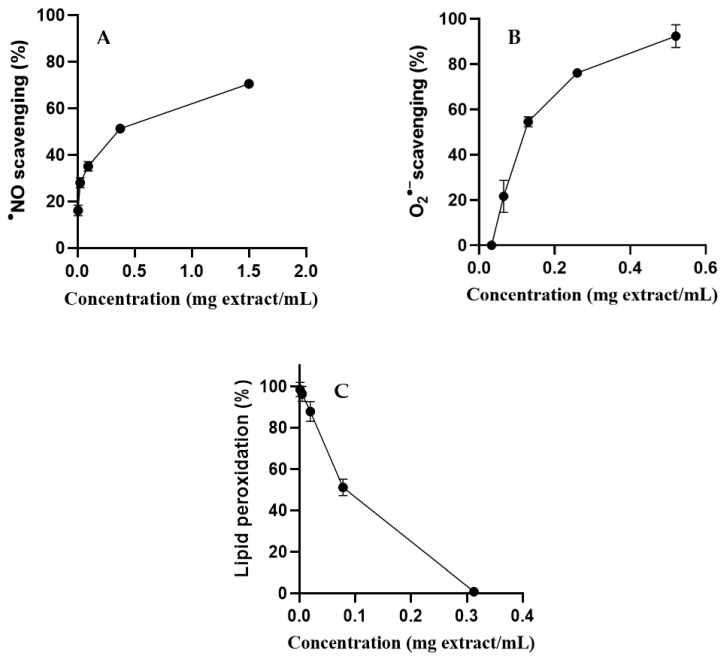
^•^NO scavenging (**A**), O_2_^•−^ scavenging (**B**) and inhibition of lipid peroxidation (**C**) by *S. betaceum* leaf aqueous extract. Results are expressed as mean ± SD of three independent experiments, each performed in triplicate.

*S. betaceum* leaf aqueous extract showed a good concentration-dependent scavenging capacity against ^•^NO (IC_50_ = 0.299 mg/mL) (Figure 4A) and O_2_^•−^ (IC_50_ = 0.119 mg/mL) (Figure 4B).

*S. betaceum* leaf aqueous extract was evaluated for its capacity to inhibit the first stages of lipid peroxidation induced by the Fe^2+^/ascorbate system following the formation of conjugated dienes in linoleic acid solutions. It was possible, at the maximum concentration of *S. betaceum* leaf aqueous extract tested (0.3 mg/mL), to completely inhibit the peroxidation of linoleic acid triggered by Fenton’s reaction (Figure 4C). The extract protected linoleic acid from the peroxidation process in a concentration-dependent manner, presenting an IC_50_ value close to that obtained for BHT (IC_50_ = 0.07 mg/mL and IC_50_ = 0.08 mg/mL, respectively). This result highlights this matrix as an interesting iron-catalyzed lipid peroxidation inhibitor. This is the first report on the scavenging activity of *S. betaceum* leaf extract against the biologically relevant radicals ^•^NO and O_2_^•−^, as well as its effect on the lipid peroxidation process.

The chemical complexity of the extract does not allow us to predict which compounds are responsible for the protective effects displayed. Moreover, the activity of the extract may not be attributed to a single compound or chemical class but rather to the existence of synergistic phenomena between the total extract constituents. Nonetheless, we suppose that the antioxidant activity observed is mainly related to its phenolic composition. Indeed, numerous studies have indicated that many of the phenolic compounds found in *S. betaceum* leaf aqueous extract have a concentration-dependent antiradical activity against nitric oxide and superoxide anion radicals and exert protective effects on linoleic acid peroxidation triggered by Fenton’s reaction [28,29]. The high degree of hydroxylation and the presence of a catechol group (*O*-dihydroxyl structure) are two structural aspects described as determinants of the enhancement of the antioxidant activity of the phenolic compounds. These features are present in the phenolic constituents of aqueous extract obtained from *S. betaceum*.

Although the capacity to neutralize free radicals is commonly used to measure the antioxidant activity of extracts or compounds, it should be highlighted that in vitro assays can only rank antioxidant activities for their particular reaction systems, and their relevance to in vivo health protective activities is uncertain. Therefore, it is prudent to use more than one type of antioxidant assay to measure antioxidant activity and to include at least one assay that has biological relevance. Although no single in vitro antioxidant activity assay can reflect the possible biological impact, herein, it was demonstrated that this extract reacts directly with biologically relevant radicals, and inhibiting lipid peroxidation may contribute to the prevention of several diabetes-related complications.

## 3. Materials and Methods

### 3.1. Standards and Reagents

Caffeic acid, rosmarinic acid, acarbose, sodium nitroprusside dihydrate (SNP), sodium phosphate, phosphoric acid (H_3_PO_4_), sulphanilamide, β-nicotinamide adenine dinucleotide in its reduced form (NADH), nitroblue tetrazolium (NBT), phenazine methosulphate (PMS), monopotassium phosphate (KH_2_PO_4_), linoleic acid, ferrous sulfate heptahydrate (FeSO_4_·7H_2_O), ascorbic acid, ethanol, *p*-nitrophenyl-α-glucopyranoside (NGP), α-glucosidase (from Saccharomyces cerevisiae), monopotassium phosphate (KH_2_PO_4_), trisodium phosphate (Na_3_PO_4_), hydrochloride acid (HCl), starch, α-amylase (porcine pancreas), dinitrosalicylic acid (DNS), sodium hydroxide (NaOH), potassium sodium tartrate tetrahydrate (KNaC_4_H_4_O_6_·4H_2_O), d,l-glyceraldehyde, β-nicotinamide adenine dinucleotide phosphate in its reduced form (NADPH) and 2,6-di-*tert*-buty-l-4-methylphenol (BHT) were acquired from Sigma–Aldrich (St. Louis, MO, USA). Human aldose reductase was obtained from Prozomix (Northumberland, UK). Tris(hydroxymethyl)aminomethane hydrochloride (Tris-HCl) was purchased from AMRESCO (Solon, Ohio, USA). *N*-(1-naphthyl) ethylenediamine dihydrochloride was obtained from Fisher Chemical (UK). 3-*O*-Caffeoylquinic acid and 4-*O*-caffeoylquinic acid were acquired from ChemFaces (Wuhan, Hubei, China). 5-*O*-Caffeoylquinic acid, kamferol-3-*O*-rutinoside, quercetin-3-*O*-rutinoside and quercitrin were acquired from Extrasynthese (Genay Cedex, France).

Water was treated using a Milli-Q water purification system (Millipore, Bedford, MA, USA).

### 3.2. S. betaceum Leaves’ Collection

Leaves from the *S. betaceum* tree were collected in June 2022 in Maceda (N 40°55′33″, W 8°36′48″) (Portugal). After collection, the vegetal material was immediately transported to the laboratory, where they were kept at −20 °C prior to their lyophilization using a Virtis SP Scientific Sentry 2.0 apparatus (Gardiner, NY, USA). The dried material was then powdered (<910 µm) and stored in the dark in a desiccator before use. The voucher specimen was deposited in Laboratório de Farmacognosia, Faculdade de Farmácia, Universidade do Porto (Porto, Portugal, LSbM-200622).

### 3.3. Aqueous Extraction

The aqueous *S. betaceum* leaf extract was prepared by decoction, following the procedure reported by Bernado et al. [28]. Briefly, ca. 3 g of powdered leaf was boiled with 500 mL of water for 30 min. The obtained aqueous extract was filtered using a Büchner funnel and lyophilized. A yield of ca. 1.018 g was obtained. The lyophilized extract was kept in a desiccator in the dark until the time of analysis. For its phenolic determination, the leaf extract was redissolved in water. For the biological assays, the extract was redissolved in water or a buffer.

### 3.4. Phenolic Compounds Profiling

#### 3.4.1. HPLC-DAD Analysis

For the phenolic compound profiling, the dried aqueous extract of *S. betaceum* leaf was redissolved in Milli-Q water, sonicated, filtered through a 0.45 μm pore membrane (Millipore, Bedford, MA, USA) and then analyzed using an analytical HPLC unit (Gilson Medical Electronics, Villiers le Bel, France) using a Spherisorb ODS2 (25.0 × 0.46 cm; 5 μm, particle size) column under the chromatographic conditions previously described by Magalhães and colleagues [30]. Spectral data from all the peaks were collected in the range of 200–700 nm, and chromatograms were recorded at 320 and 350 nm for hydroxycinnamic acid and flavonoid quantification, respectively. The data were processed using Unipoint System software (Gilson Medical Electronics, Villiers le Bel, France). The compounds were identified by comparing their retention times and UV–Vis spectra in the range of 200–700 nm with those of authentic standards injected under the same chromatographic conditions. The peak purity was checked using the software contrast facilities.

For quantification purposes, 20 μL of redissolved aqueous extract was injected in triplicate. Phenolic compound quantification was achieved by obtaining the absorbance recorded on the chromatograms relative to external standards. All compounds were quantified as themselves, except for 3-*O*-caffeoylquinic acid (**1**), which was quantified as 4-*O*-caffeoylquinic acid (**2**), and the unknown kaempferol glycoside derivatives (**6** and **9**) and unknown hydroxycinnamic acid derivatives (**7** and **8**) were determined as kaempferol-3-*O*-rutinoside and 5-*O*-caffeoylquinic acid, respectively.

##### Linearity

**Figure 5:**
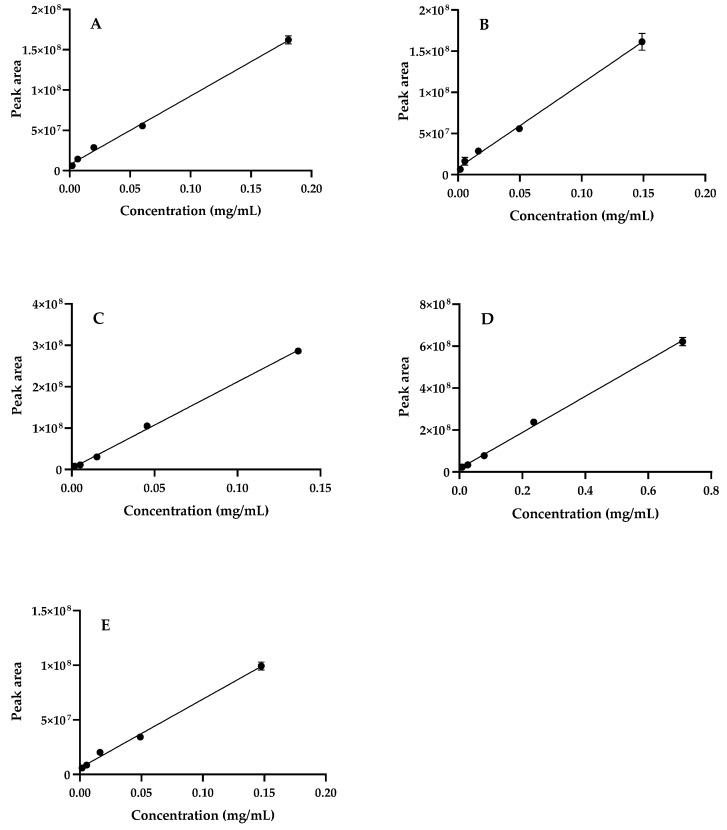
Calibration curves of (**A**) 4-*O*-caffeoylquinic acid; (**B**) chlorogenic acid; (**C**) caffeic acid; (**D**) rosmarinic acid; (**E**) kaempferol-3-*O*-rutinoside; Regression equation and *r*^2^ values are presented in Table 1. Results are expressed as mean ± SD of three independent analysis.

The linearity range of the method was assessed by building calibration curves using five different concentration levels of the pure standards, according to the range of concentrations present in the sample (Figure 5).

### 3.5. Biological Assays

#### 3.5.1. α-Amylase Inhibition

The capacity of *S. betaceum* aqueous extract to inhibit the activity of α-amylase was evaluated by measuring the 3-Amino-5-nitrosalicylic acid (ANS) that was formed in the reaction between α-amylase and starch with DNS at 540 nm, mimicking the procedure previously reported by Valentão and colleagues [31]. The inhibitory activity was calculated using the following formula: α-amylase inhibition (%) = [1 − (A extract − A blank)/(A control − A blank)] × 100%, where A corresponds to the absorbance. Three independent experiments were performed in triplicate. The pharmacological inhibitor acarbose was selected as the positive control.

#### 3.5.2. α-Glucosidase Inhibition

The capacity of *S. betaceum* aqueous extract to inhibit α-glucosidase was evaluated according to a previously reported procedure [17]. The inhibition of α-glucosidase activity was calculated as follows: α-glucosidase inhibition (%) = [1 − (A extract/A control)] × 100%], where A is the absorbance. Three independent experiments were performed in triplicate. Acarbose was selected as the positive control.

#### 3.5.3. Aldose Reductase Inhibition

The potential of *S. betaceum* aqueous extract to inhibit aldose reductase was evaluated by measuring the NADPH rate consumption, following the procedure reported Ferreres and co-workers [17]. The following formula was used to calculate the inhibitory activity: aldose reductase inhibitory activity (%) = 100 × (1 − [A extract/A control]), with A being the absorbance measured. Six independent experiments were performed in triplicate. Rutin was used as the positive control.

#### 3.5.4. Lipid Peroxidation

The peroxidation of linoleic acid was determined according to the formation of conjugated dienes, as mentioned by Bernardo and colleagues [28]. The results were expressed as the extent of lipid peroxidation following the equation: lipid peroxidation (%) = 100 × [(absorbance of sample − absorbance of blank)/(absorbance of control − absorbance of blank)]. Three independent experiments were performed in triplicate. BHT was used as the positive control.

#### 3.5.5. Superoxide Anion Radical Scavenging

The capacity of *S. betaceum* aqueous extract to scavenge O_2_^•−^ was determined using a non-enzymatic system (NADH/PMS system), as described by Lopes and co-workers [31]. The scavenging capacity was calculated using the following equation: O_2_^•−^ scavenging (%) = 100 × [1 − (A extract/A control)], where A corresponds to the absorbance measured. Three independent experiments were performed in triplicate. Quercetin was used as the positive control.

#### 3.5.6. Nitric Oxide Radical Scavenging

The capacity of *S. betaceum* leaf extract to scavenge ^•^NO was determined by the Griess reaction, according to Pereira and colleagues [32]. The scavenging activity was calculated using the following equation: ^•^NO scavenging (%) = 100 × [1 − (A extract − A blank)/(A control − A blank)], with A being the absorbance measured. Three independent experiments were performed in triplicate. Quercetin was used as the positive control.

#### 3.5.7. Data Processing

Data analysis was performed using GraphPad Prism 8 software (San Diego, CA, USA) for Windows.

## 4. Conclusions

Herein, the phenolic profile and the antidiabetic potential of *S. betaceum* leaves was disclosed for the first time. The *S. betaceum* leaf aqueous extract revealed a phenolic profile mainly composed of hydroxycinnamic acids, with rosmarinic acid being the predominant compound. The extract displayed inhibitory activity towards α-glucosidase, showing the potential to positively contribute to glycaemia control. Nevertheless, *S. betaceum* leaf aqueous extract showed to be most promising in the management of diabetes-related complications:the aqueous extract showed a high inhibitory capacity towards aldose reductase, a potent capacity to intercept the in vitro-generated reactive species O_2_^•−^ and ^•^NO and a strong ability to completely protect linoleic acid from the peroxidation process. Previous data allow us to conclude that the antidiabetic effects of the extract are likely related to its phenolic composition. The biological potential of *S. betaceum* leaves revealed by our work opens the door for the use of unexplored species and those at high risk of extinction.

## Figures and Tables

**Table 1:** Phenolic compound contents of *S. betaceum* aqueous extract. Regression equation, *r*^2^ values and linearity of the reference compounds with the employed analytical conditions.

Peak	Compound	Regression Equation (mg/mL)	*r* ^2^	Linearity (mg/mL)	*S. betaceum* Leaves (mg/g Dry Extract) ^1^
**1**	3-*O*-Caffeoylquinic acid	*y* = 8.5 × 10^8^*x* + 7,222,443.7	0.997	0.002–0.181	2.91 ± 0.21
**2**	4-*O*-Caffeoylquinic acid	*y* = 8.5 × 10^8^*x* + 7,222,443.7	0.997	0.002–0.181	3.57 ± 0.10
**3**	5-*O*-Caffeoylquinic acid	*y* = 1.0 × 10^9^*x* + 8,240,529.4	0.997	0.002–0.149	5.09 ± 0.01
**4**	Caffeic acid	*y* = 2.1 × 10^9^*x* + 3,056,465.2	0.999	0.002–0.136	0.81 ± 0.01
**5**	Rosmarinic acid	*y* = 8.6 × 10^8^*x* + 16,145,014.1	0.998	0.009–0.709	99.62 ± 1.02
**6 + 9**	Unknown kaempferol glycoside deriv.	*y* = 6.3 × 10^8^*x* + 5,924,781.1	0.996	0.002–0.148	7.75 ± 0.22
**7 + 8**	Unknown hydroxycinnamic acid deriv.	*y* = 1.0 × 10^9^*x* + 8,240,529.4	0.997	0.002–0.149	1.82 ± 0.05
	Total				121.57 ± 1.62

^1^ Results are expressed as the mean ± standard deviation of three determinations. “Deriv.”—derivatives.

## Data Availability

Not applicable.

## Sample Availability

Samples of the compounds are not available from the authors.
